# Regulation of PLC*β*_2_ by the electrostatic and mechanical properties of lipid bilayers

**DOI:** 10.1038/srep12628

**Published:** 2015-08-05

**Authors:** Alessia Arduin, Piers R. J. Gaffney, Oscar Ces

**Affiliations:** 1 Institute of Chemical Biology, Department of Chemical Biology, Imperial College London, Exhibition Road, London SW7 2AZ, United Kingdom

## Abstract

Phosphoinositide-specific phospholipase C (PLC) is an important family of enzymes constituting a junction between phosphoinositide lipid signaling and the trans-membrane signal transduction processes that are crucial to many living cells. However, the regulatory mechanism of PLC is not yet understood in detail. To address this issue, activity studies were carried out using lipid vesicles in a model system that was specifically designed to study protein-protein and lipid-protein interactions in concert. Evidence was found for a direct interaction between PLC and the GTPases that mediate phospholipase activation. Furthermore, for the first time, the relationships between PLC activity and substrate presentation in lipid vesicles of various sizes, as well as lipid composition and membrane mechanical properties, were analyzed. PLC activity was found to depend upon the electrostatic potential and the stored curvature elastic stress of the lipid membranes.

Phosphoinositide-specific phospholipase C (PLC) is an intensively studied family of enzymes that regulates important cellular processes through their pivotal roles in trans-membrane signal transduction and phosphoinositide lipid signaling^1^,^2^,^3^. PLCs catalyze the hydrolysis of phosphatidylinositol 4,5-diphosphate [PtdIns(4,5)P_2_] to diacylglycerol and *myo*-inositol 1,4,5-triphosphate (IP_3_), which are both well known intracellular second messengers. PLCs are activated in response to the stimulation of cell surface receptors at the plasma membrane, after which the signals are carried downstream by other transducers, such as small GTPases. GTPases bound to guanosine triphosphate (GTP) induce translocation of PLC from the cytosol to the plasma membrane where the substrate of PLC is localized. However, this PLC-GTPase interaction is thought to be insufficient to cause full PLC activation. Instead, it has been proposed that a concomitant PLC-GTPase and PLC-lipid interaction is necessary to fully activate PLC, as described by the auto-inhibitory model (Fig. 1)^4^. According to this model, the linker between the X and Y halves of the PLC catalytic domain occludes the catalytic site. Upon binding to the GTPase, PLC translocates to the plasma membrane. It also changes conformation due to charge repulsion between the negatively charged X-Y linker and the negatively charged plasma membrane, augmented by steric compression between the X-Y linker and the membrane interface. As a result of this conformational rearrangement, the catalytic pocket of PLC finally becomes accessible to its PtdIns(4,5)P_2_ substrate^4^.

Initially the auto-inhibitory model was based on structural information and activity data from PLC*β*_2_, but it was then extended to other PLC subfamilies based on further activity data and sequence similarities^4^. Indeed, mutants of PLC*β*_2_ with the X-Y linker destabilized (G530P), or partially deleted (∆516–537 AA, ∆470–515 AA or ∆470–524 AA), showed higher basal activity than the wild type (w.t.). In addition, activation was unaltered in the presence of transducers such as Rac3, G*β*_1_*γ*_1_ or G*α*q, thereby excluding a role for the X-Y linker in the interaction between PLC and the transducer^4^. Similar activity modulation was observed for other PLC subfamilies, such as PLC*δ* and PLC*ε*. Sequence analysis of the X-Y linker across PLC members highlights clusters of negatively charged residues (PLC*η*_1_, PLC*η*_2_), or an excess of negatively charged residues (PLC*β*, PLCε). Furthermore, studies of PLC*ζ* indicated that PLC activation here resulted from protein-membrane anchoring, mediated by electrostatic interactions between the basic cluster of residues within the X-Y linker and acidic lipids such as PtdIns(4,5)P_2_^5^. Taken together, these reports on various members of the PLC family suggested that the properties of the lipid membrane play an important role in PLC regulation.

In order to elucidate the mechanism of activation and regulation of PLC with respect to the auto-inhibitory mechanism, and the possible regulatory role played by membrane properties, we investigated the dependency of PLC activity on lipid presentation. PLC*β*_2_ was adopted as our model protein and its activity was measured using a flexible cell-free system. In this system the lipid presentation was tightly controlled, reconstituting the PLC-substrate interaction and GTPase-PLC activation *in vitro*^6^. PLC activity and its activation by interaction with a GTPase (in a guanine nucleotide dependent manner) were monitored as functions of the membrane curvature, their electrostatic interactions, and a mechanical membrane property known as the stored curvature elastic stress^7^,^8^,^9^. These membrane properties are defined by the lipids within the bilayer, and therefore they can be varied by changing the lipid composition of the bilayer used in the cell-free system.

Stored curvature elastic stress was modulated by varying the proportion of phosphatidylethanolamine (PE) within the lipid bilayer. PE is a so-called ‘non-bilayer’ lipid that in the absence of physical constraints would tend to form monolayers with negative curvature (towards the polar region). When such monolayers are coupled together in a bilayer arrangement the monolayers still want to curve away from each other, but the formation of such a structure is not energetically favorable, not least because it would result in vacuum regions. Thus, the tendency of PE-rich lipid leaflets to curve is frustrated, with opposing layers pinned against each other, giving rise to lamellar bilayers having high levels of stored curvature elastic stress. Several studies have shown that stored curvature elastic stress can regulate the activity of membrane proteins or peripheral proteins^10^, as has been demonstrated for phospholipase A_2_^11^, CTP: phosphocholine cytidylyltransferase^12^,^13^ and protein kinase C^14^,^15^. To investigate if PLCs could also belong to this group of membrane sensing proteins, the effects of electrostatic potential and of curvature elastic stress upon protein-membrane interactions were decoupled and investigated separately.

## Methods

### Prenylation of GTPases *in vitro*

Rac2, being a GTPase of the RhoA subfamily, was prenylated *in vitro* by GGTase type I. The Rac2 prenylation reaction mixtures (20 *μ*l final volume) were prepared in glass tubes containing: purified wild-type RhoA (5 *μ*M), geranylgeranylpyrophosphate (5 mM) and 50 nM of GGTase type I. The reaction samples were incubated for 30 min at 37 °C, and stored on ice for 10 min prior to addition to the cell-free reaction mixtures.

### Lipid vesicle preparation

Lipid stock solutions were pipetted into a 14 × 100 mm Pyrex glass tube to obtain 33.4 *μ*M PtdIns(4,5)P_2_/[^3^H]-PtdIns(4,5)P_2_ (185 GBq/mol) and 536 *μ*M final concentrations of other lipids. The lipid mixture was evaporated under a stream of nitrogen, then re-suspended in the appropriate buffer (20 *μ*l per assay) by vortexing for 30 min. The mixtures were sonicated for 5 min, 1% amplitude, 4 °C in a cup horn-type sonicator (XL Ultrasonic Processor, Misonix), or extruded 31 times across a polycarbonate membrane (Nuclepore track-etched membranes, Whatman) of a defined pore size (50, 100 and 400 nm) using a mini extruder (Avanti Polar Lipids). The lipid vesicles were immediately used for the reconstitution assay.

### PLC activity measurements

The cell-free assay was performed for specific protein concentrations, and with free Ca^2+^ concentrations that were calculated using the EqCal (Biosoft) software. The PLC was diluted in the presence of BSA to minimize non-specific binding of the PLC to the tubes. All the samples were prepared with identical free Ca^2+^ concentration (300 nM), except for those samples used to determine the maximum activity. The latter samples were prepared with a higher free Ca^2+^ concentration (100 *μ*M) plus sodium deoxycholate (NaDC, 2 mM). Each set of samples was prepared in duplicate. The reaction samples (60 *μ*l final volume) contained: 50 mM Tris maleate pH 7.3, 70 mM KCl, 3 mM ethylene glycol tetraacetic acid (EGTA), 2 mM DTT, 3.6 mM GTP*γ*S or guanosine diphosphate (GDP) (10 *μ*l), a defined free Ca^2+^ concentration, either GTPase in membrane solubilization buffer from Sf9 (5 *μ*l), or *in vitro* prenylated GTPase (5 *μ*l). In the set of samples for determining the basal activity, the GTPase was substituted with GTPase buffer. The blank was prepared without PLC. The reaction was started by the addition of the substrate-containing mixed lipid vesicles (20 *μ*l). The reactions were incubated for 45 min at 30 °C, then terminated by the addition of ice-cold CHCl_3_-MeOH-conc. HCl (100:100:0.6 v/v, 350 *μ*l), followed by the addition of 1 M HCl and 5 mM EGTA (100 *μ*l). The samples were kept for 10 min on ice, then spun at 20,800 *g* for 1 min in a tabletop centrifuge (5417R, Eppendorf) to improve the phase separation. An aliquot of the aqueous phase (200 *μ*l), which contained the water-soluble product IP_3_, was pipetted into a scintillation tube and mixed with scintillation fluid (4 ml, Ultima Gold, Perkin Elmer). The samples were vortexed and loaded into a scintillation counter (Liquid scintillation analyzer 2500TR, Packard).

### Small angle X-ray scattering

The lipid mixtures were lyophilized at −55 °C under high vacuum, hydrated in 70% w/w UF water, or cell-free assay buffer, and then homogenized by five freeze-thawing cycles. The lipid mixtures were transferred into a SAXS capillary (1.5 mm in diameter), which was then sealed under a flame to prevent evaporation. Each sample was placed in a beam line, built in-house, equipped with an X-ray generator with copper target and a nickel filter (Bede microsource) and exposed 4 × 60 sec. at 30 °C. The X-ray diffraction images were acquires on a Gemstar HS intensified CCD detector (Photonic Science) and the spectra obtained were analyzed using the software Axcess in order to calculate the *d* spacing and determine the lipid phase.

## Results

### PLC*β*_2_ activity assay

**Figure 2:**
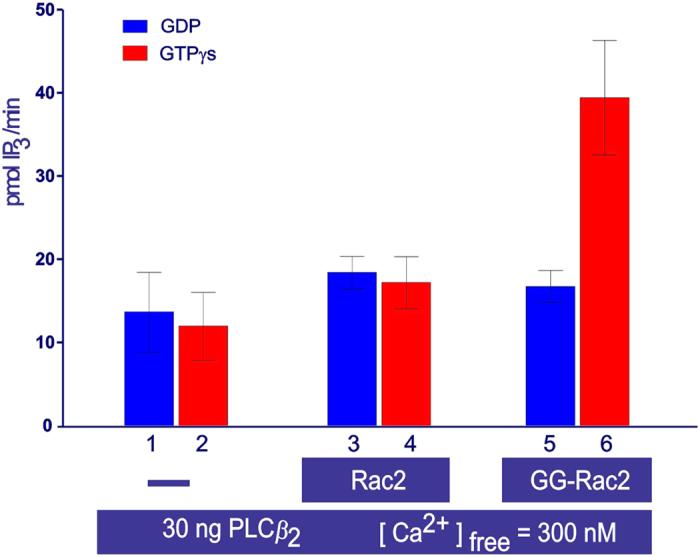
Activation of PLC*β*_2_ by GTP bound prenylated Rac2 in the cell-free system. The bar chart reports the average activity measured in duplicate samples (± the s.e.m.) upon sample incubation at 30 °C for 45 min. The samples contained: purified PLC*β*_2_ (2–803 AA) (0.5 ng/μl, equivalent to 5.4 nM), DOPE: PtdIns (4,5)P_2_/[^3^H]-PtdIns (4,5)P_2_ lipid vesicles 16:1 mol/mol, 300 nM free calcium, 100 μM GDP (blue bars), 100 μM GTP*γ*S (red bars), and 1.6 ng/μl GG-Rac2.

**Figure 3:**
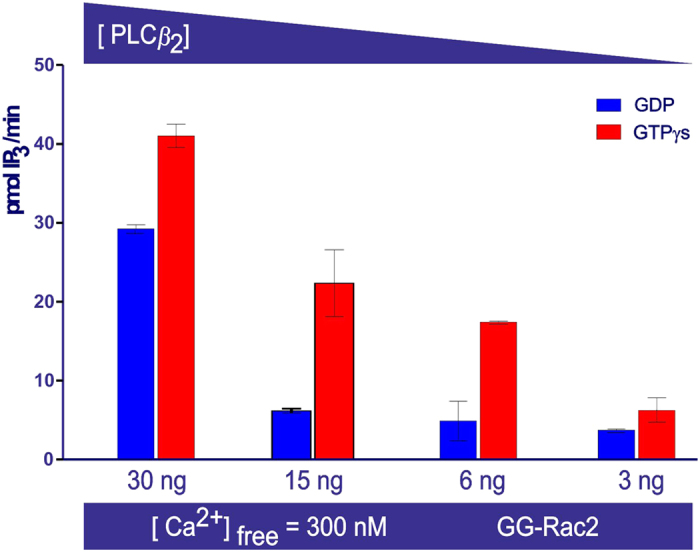
The response curve of PLC*β*_2_ (2–803 AA) using the cell-free system. The bar chart shows the average activity measured in duplicate samples (± the s.e.m.). Different concentrations of PLC*β*_2_ were tested (30 ng, 15 ng, 6 ng or 3 ng, in 60 μl final sample volume), but all other conditions are the same as for Fig. 2.

Control experiments showed that the basal activity of PLC*β*_2_ (2–803 AA) in the absence of GTPase was unaffected by the presence of either GDP or GTP*γ*S (columns 1 and 2, Fig. 2), and that the addition of un-prenylated Rac2 to the cell-free system caused a negligible increase in PLC activity. This did not change in the presence of GDP (Fig. 2, columns 1 and 3) or of GTP*γ*S (Fig. 2, columns 2 and 4). When geranylgeranylated Rac2 (GG-Rac2) was added, the PLC activity measured in the presence of GDP was again comparable to the activity measured in the presence of GDP plus un-prenylated Rac2. However, in the presence of GTP*γ*S, PLC activity was 3-fold higher than that measured in the presence of GDP and GG-Rac2 (Fig. 2, columns 5 and 6). The response curve shows that the range of concentrations of PLC*β*_2_ (2–803 AA) tested were in the linear range (Fig. 3).

### PLC*β*_2_ activity as a function of membrane curvature

**Figure 4:**
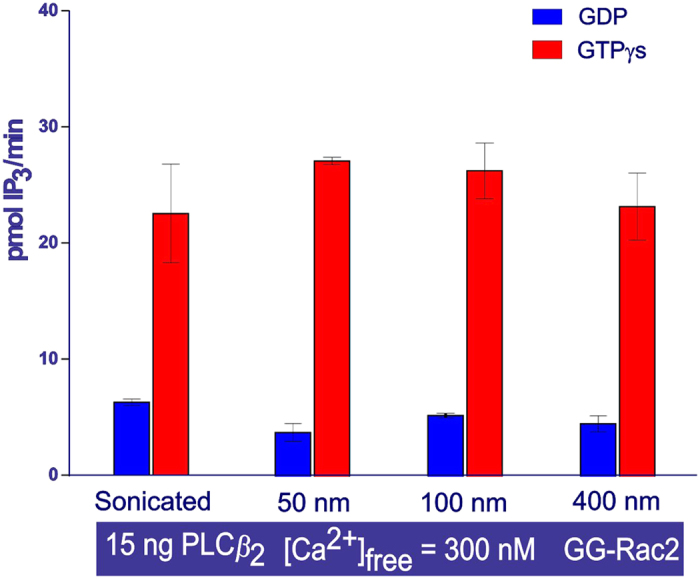
The effect on PLC*β*_2_ activity of lipid bilayer curvature. The bar chart shows the average activity measured in duplicate samples (± the s.e.m.). The activity was monitored using the cell-free system and with lipid vesicles of different sizes. The lipid vesicles were prepared by extrusion using various pore size filters (50, 100, 400 nm) or by sonication. The samples contained: purified PLC*β*_2_ (0.25 ng/μl, equivalent to 2.7 nM). All other conditions are the same as for Fig. 2.

Lipid vesicles (DOPE: PtdIns(4,5)P_2_/[^3^H]-PtdIns(4,5)P_2_ 16:1 mol/mol) of different diameters (50, 100 and 400 nm), and therefore of different membrane curvatures, were obtained by extrusion and their normal distribution was confirmed by dynamic light scattering. The data generated at this stage showed no significant variation in PLC*β*_2_ activity in the presence of GDP and GG-Rac2, and no variation in PLC*β*_2_ (2–803 AA) stimulation (3-fold) in the presence of GTP*γ*S and GG-Rac2 using lipid vesicles of different membrane curvature (Fig. 4).

### PLC*β*_2_ activity as a function of membrane charge

**Figure 5:**
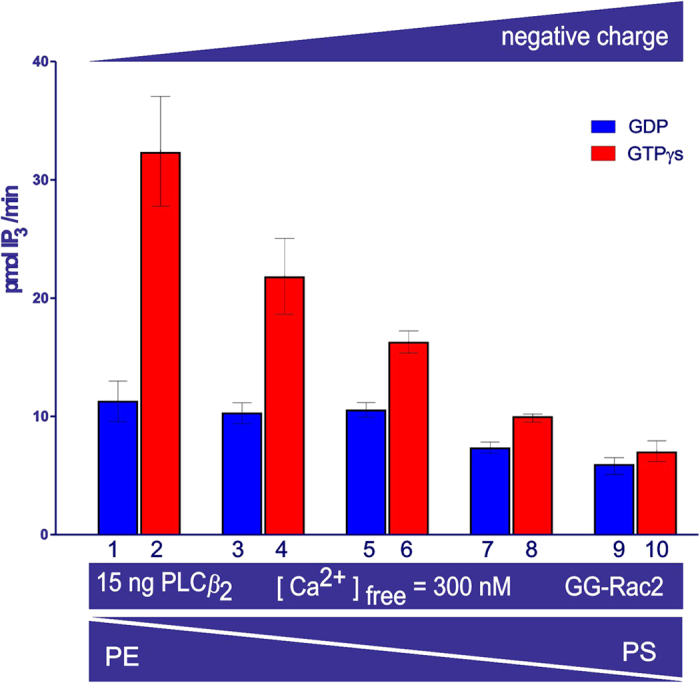
The effect on PLC*β*_2_ activity of negatively charged lipid vesicles. The bar chart shows the average activity measured in duplicate samples (± the s.e.m.). The lipid vesicles have the following DOPE: DOPS ratios: 16:0 (columns 1 & 2), 11.2:4.4 (columns 3 & 4), 8.0:7.3 (columns 5 & 6), 4.8:10.3 (columns 7 & 8) and 0:16 (columns 9 & 10) mol/mol. The proportion of PtdIns (4,5)P_2_ substrate in the vesicles was constant at (DOPE + DOPS): PtdIns (4,5)P_2_/[^3^H]-PtdIns (4,5)P_2_ 16:1 mol/mol. All other conditions are the same as for Fig. 2.

**Figure 6:**
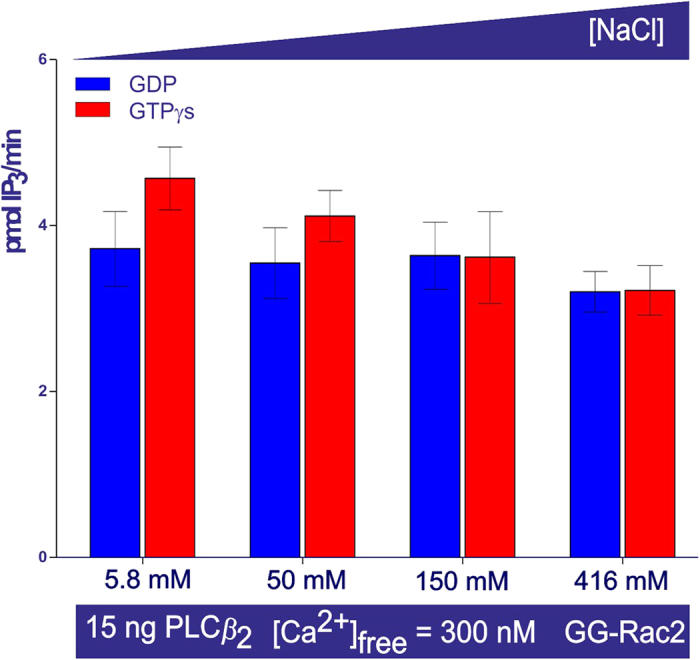
Effect on PLC*β*_2_ activity of charge shielding. The bar chart shows the average activity measured in duplicate samples (± the s.e.m.). The lipids and PLC surface charges were progressively more shielded by increasing the concentration of NaCl over two orders of magnitude, with lipid vesicles containing DOPE: DOPS: PtdIns (4,5)P_2_/[^3^H]-PtdIns (4,5)P_2_ 4.8:10.3:1 mol/mol, but had little effect on PLC activity.

Upon stimulating PLC*β*_2_ (2–803 AA) with GTP*γ*S and GG-Rac2, the activity decreased as the negative charge on the lipid vesicles increased (see Fig. 5, columns 2, 4, 6, 8 and 10), and PLC activity was not restored in the presence of increasing concentrations of NaCl (Fig. 6, from 5.8 mM to 416 mM). By contrast there was no significant change in PLC activity in the presence of GDP and GG-Rac2 (Fig. 5, columns 1, 3, 5, 7 and 9), or with negatively charged lipid vesicles over the same range of NaCl concentrations (Fig. 6).

Non-radiolabeled lipid mixtures of *sn*-1,2-dioleoyl-phosphatidylethanolamine (DOPE): *sn*-1,2-dioleoyl-phosphatidylserine (DOPS): PtdIns(4,5)P_2_ (11.2:4.4:1 and 8.0:7.4:1 mol/mol), similar to the lipid mixtures used for lipid vesicle preparation, were analyzed by small angle X-ray scattering (SAXS) in order to characterize their lipid phase. Each lipid mixture tested was found to be in a fluid lamellar phase (Table 1).

### PLC*β*_2_ activity as a function of the stored curvature elastic stress

**Figure 7:**
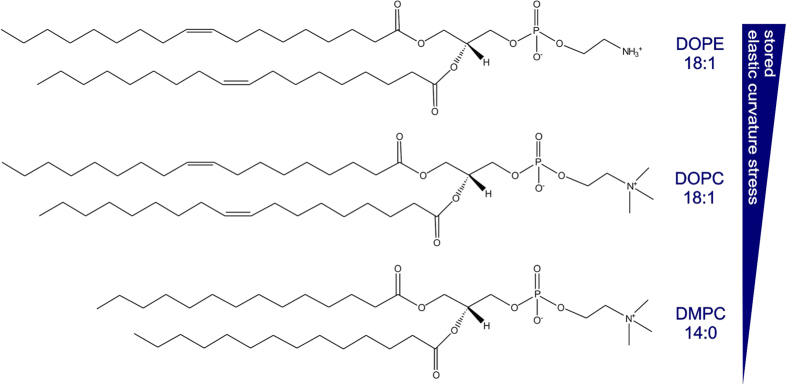
Molecular structures of DOPE, DOPC and DMPC used to prepare the lipid vesicles adopted in the cell-free system to investigate the curvature elastic stress effect on PLC*β*_2_ activity.

The PLC*β*_2_ (2–803 AA) activity was modulated as a function of the stored curvature elastic stress within the lipid vesicles by changing the lipid composition (Fig. 7). The stored curvature elastic stress was first decreased by substituting DOPE with *sn*-1,2-dioleoyl-phosphatidylcholine (DOPC); the lower hydrophilicity and inability to hydrogen-bond of the trimethylammonium moiety reduces attraction between DOPC head-groups. Curvature elastic stress was then decreased further by substituting DOPC with *sn*-1,2-dimyristoyl-phosphatidylcholine (DMPC); both shortening the acyl chains from C_18_ to C_14_, and removing their unsaturation, reduce the volume fraction of the fatty acid tails.

**Figure 8:**
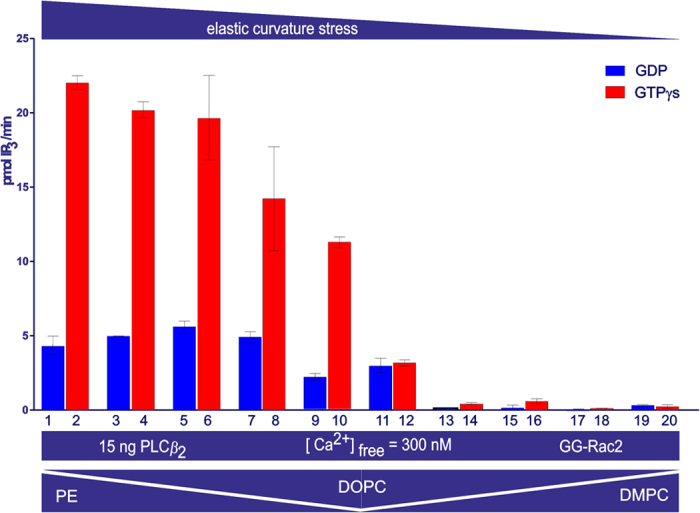
Effect on PLC*β*_2_ activity of curvature elastic stress. The bar chart shows the average activity measured in duplicate samples (± the s.e.m.). The lipid vesicles tested for each set of samples have the following lipid ratios: DOPE:DOPC 16:0 (columns 1 & 2), 12.8:2.8 (columns 3 & 4), 11.1:4.2 (columns 5 & 6), 8.0:7.0 (columns 7 & 8), 4.8:9.8 (columns 9 & 10) and 0:16 (columns 11 & 12) mol/mol, and then DOPC:DMPC 9.8:4.9 (columns 13 & 14), 7.0:8.1 (columns 15 & 16), 4.2:11.4 (columns 17 & 18) and 0:16 (columns 19 & 20) mol/mol. The proportion of PtdIns (4,5)P_2_ substrate in the vesicles was constant at (DOPE + DOPC + DMPC): PtdIns (4,5)P_2_/[^3^H]-PtdIns (4,5)P_2_ 16:1 mol/mol. All other conditions are the same as for Fig. 2.

Upon interaction with GG-Rac2 the stimulation of PLC*β*_2_ (2–803 AA) decreased with the fall in the proportion of PE and the corresponding rise in that of DOPC (from DOPE: PtdIns(4,5)P_2_/[^3^H]-PtdIns(4,5)P_2_ 16:1 mol/mol, to DOPC: PtdIns(4,5)P_2_/[^3^H]-PtdIns(4,5)P_2_ 16:1 mol/mol vesicles). The decrease of PLC*β*_2_ stimulation was even more pronounced, and then disappeared, when the DOPC in the lipid vesicles was replaced by DMPC (Fig. 8). These data indicated that the lower the stored curvature elastic stress in the lipid vesicles, the lower the activity of PLC in the presence of GTP*γ*S and GG-Rac2 (PLC*β*_2_ stimulation).

The lipid phases of the DOPE: DOPC: PtdIns(4,5)P_2_ (11.1:4.2:1, 8.0:7.0:1 and 4.8:9.8:1 mol/mol) and of the DOPC: DMPC: PtdIns(4,5)P_2_ (9.8:4.9:1, 7.0:8.1:1 and 4.2:11.4:1 mol/mol) lipid mixtures were determined by SAXS and all were found to have the characteristic 1:2:3:4 ring ratio of the fluid lamellar lipid phase regardless of whether they had been hydrated in ultra-filtered (UF) water, or in cell-free assay buffer (Table 1).

### The function of the X-Y linker in the regulatory mechanism of PLC*β*_2_

**Figure 9:**
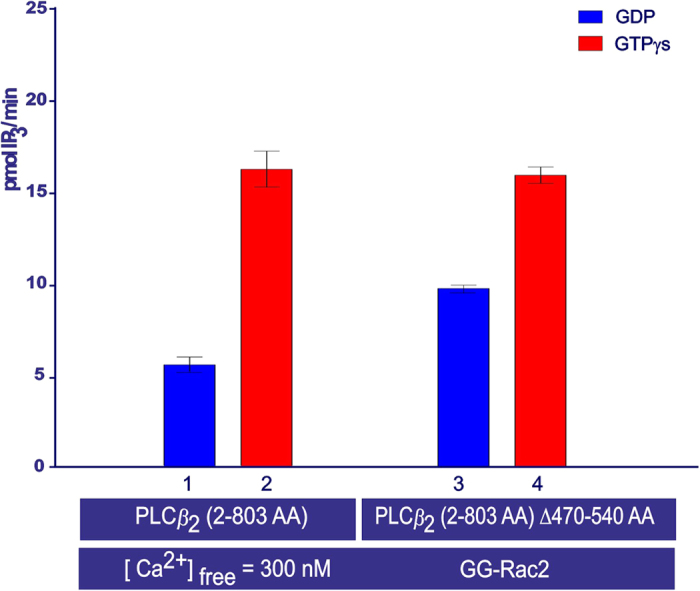
PLC*β*_2_ activity measurements obtained using the cell-free system. The bar chart shows the average activity after sample incubation at 30 °C for 45 min, measured in duplicate samples (± the s.e.m.). The samples contained: purified w.t. PLC*β*_2_ (2–803 AA) (0.25 ng/μl), or purified PLC*β*_2_ (2–803 AA) ∆(470–540) (0.125 ng/μl), and DOPE: PtdIns (4,5)P_2_/[^3^H]-PtdIns(4,5)P_2_ lipid vesicles 16:1 mol/mol. All other conditions are the same as for Fig. 2.

We tested different concentrations of PLC*β*_2_ (2–803 AA) ∆(470–540), in which the X-Y linker had been deleted, to find values that gave a similar response to w.t. PLC*β*_2_ (2–803 AA) in the cell-free system. In the presence of GTP*γ*S and GG-Rac2, only 1.5 nM concentration of PLC*β*_2_ (2–803 AA) ∆(470–540) (0.125 ng/*μ*l) was required to obtain a stimulation response similar to that from 2.7 nM w.t. PLC*β*_2_ (2–803 AA) (0.25 ng/*μ*l) (Fig. 9, columns 2 and 4). Furthermore, in the presence of GDP and GG-Rac2, the activity of w.t. PLC*β*_2_ (2–803 AA) was lower than the activity of PLC*β*_2_ (2–803 AA) ∆(470–540) (Fig. 9, columns 1 and 3).

## Discussion

### The cell-free system

A cell-free system was successfully used to explore how the stimulation of w.t. PLC*β*_2_ (2–803 AA) varied as a function of Rac2 concentration. The cell-free PLC assay, conducted using purified PLC*β*_2_ (2–803 AA) and purified Rac2 (subsequently prenylated *in vitro* to GG-Rac2), showed that geranylgeranylation of Rac2 is essential for PLC*β*_2_ (2–803 AA) activation and confirmed that only GTP*γ*S bound (but not GDP bound) Rac2 stimulates PLC*β*_2_ (2–803 AA)^6^,^16^. The 3-fold stimulation of PLC*β*_2_ (2–803 AA) by GTP*γ*S and GG-Rac2 measured here was in line with previously reported data^6^.

The response curve indicated that a concentration of 0.25 ng/*μ*l of PLC*β*_2_ (2–803 AA) was optimal for the experiments aiming to test the auto-inhibitory model, since both an increase and a decrease in PLC stimulation could be reliably detected. In addition, this specific design of cell-free system allowed us to vary at will the concentrations of PLC*β*_2_, PtdIns(4,5)P_2_, and free Ca^2+^, the key parameters of the system. This degree of control guaranteed very high reproducibility of the data and proved that the cell-free system was suitable not only for the study of protein-protein (PLC*β*_2_-Rac2), but also protein-lipid [PLC*β*_2_-PtdIns(4,5)P_2_] interactions. In other words, this specific experimental design was essential to extend the investigation of PLC*β*_2_’s mechanism of regulation beyond protein-protein interaction to protein-lipid interaction.

### PLC*β*_2_ activity as a function of the membrane curvature

During activation PLCs are thought to insert the hydrophobic rim of their catalytic domain into the membrane^17^. In addition, it has been shown that membrane insertion of prenylated proteins, such as a geranylgeranyl-modified protein^18^, is sensitive to membrane curvature^19^. On the basis of these observations, the possibilities that PLC*β*_2_ could sense membrane curvature, and that PLC*β*_2_ catalytic activity might be dependent on membrane curvature, were investigated. The cell-free system was used to analyze PLC*β*_2_ activity as a function of membrane curvature, using lipid vesicles of different sizes (50, 100 and 400 nm in diameter)^20^,^21^,^22^. PLC*β*_2_ activity in the presence of either GDP or GTP*γ*S with GG-Rac2 was very similar over the range tested, regardless of the size of the vesicles and corresponding membrane curvature of the lipid vesicles (Fig. 4). It was therefore reasonable to believe that there is little dependency of PLC*β*_2_ activity on membrane curvature.

### PLC*β*_2_ activity as a function of the membrane charge

The overall surface charge of lipid vesicles affects the stimulation of PLC*β*_2_ (2–803AA) by Rac2. Indeed, PLC*β*_2_ stimulation decreased progressively and eventually disappeared as the negative charge on the lipid vesicles was increased by substituting zwitterionic DOPE with anionic *sn*-1,2-dioleoyl phosphatidylserine (DOPS, Fig. 5), and the stimulation was not restored by decreasing the effect of the electrostatic potential through raising the ionic strength of the solution (Fig. 6). However, neither the overall surface charge of the lipid vesicles, nor the electrostatic potential significantly affected PLC activity in the presence of GDP and GG-Rac2 (Fig. 6).

The above data are mainly consistent with the auto-inhibitory regulatory mechanism advanced by Hicks^4^, but not entirely. According to the auto-inhibitory mechanism of PLC action, an increase in the overall negative charge of the lipid vesicles was expected to enhance the charge repulsion between the X-Y auto-inhibitory linker of PLC*β*_2_ and the lipid membrane, thereby enhancing the phospholipase activity. In other words, stimulation of the PLC*β*_2_ was expected to be directly proportional to the overall negative charge. However, the activity data presented here showed a threshold (DOPE: PtdIns(4,5)P_2_/[^3^H]-PtdIns(4,5)P_2_ 16:1 mol/mol vesicles) above which a further increase of the negative charge resulted in a decrease of PLC*β*_2_ stimulation. Therefore, it was thought that other processes, together with charge repulsion between the linker and the membrane, might play a role in the PLC*β*_2_ mechanism of activation.

In light of this, the lipid-PLC interaction was decomposed into two major components: an electrostatic and a mechanical component. The above changes in ionic strength altered the balance between the electrostatic and the hydrophobic effects, making the latter prevail over the former. Up to this point it had not been possible to determine if additional regulation was significant, once partial insertion of the PLC*β*_2_ protein into the membrane had occurred. This possibility was analyzed by changing the stored curvature elastic stress of the vesicles.

### PLC*β*_2_ activity as function of the stored curvature elastic stress

There are several hypotheses that aim to explain how non-bilayer lipids affect the membrane and the protein-membrane interaction^23^,^24^,^25^,^26^. A general model, based on the lateral pressure *π*, has been developed. The lateral pressure is related to the curvature elastic stress by the torque tension *τ*:


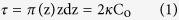


where: *κ* is the mean curvature modulus, z is the depth in the lipid bilayer, and C_o_ is the spontaneous curvature. The stored curvature elastic stress (g_c_) of a frustrated bilayer is quantified by the following function:


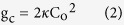


where: *κ* is the mean curvature modulus and C_o_ is the spontaneous curvature.

At equilibrium the overall lateral pressure must sum to zero, or else the bilayer would have to deform until this condition was achieved. Insertion of a peripheral membrane protein into outer leaflet of the lipid bilayer affects the lateral pressure. As a consequence of the insertion event, the lateral pressure at the lipid head-groups increases, but the lateral pressure deeper down amongst the acyl chains decreases. In the case of a membrane leaflet rich in PE, this translates into a partial release of the stored curvature stress, and the binding event is therefore favorable.

The stored curvature elastic stress may be altered by changing the composition of the lipid vesicles used in the activity assay (Table 2). In this study the stored curvature elastic stress was firstly decreased by substituting DOPE with DOPC. The latter lipid has a larger head group than DOPE and forms fewer hydrogen bonds per lipid molecule than DOPE. The stored curvature elastic stress was then further decreased by replacing the *cis*-unsaturated oleoyl fatty acid esters with myristoyl chains, substituting DOPC for DMPC, thereby reducing the acyl chain splay (Fig. 7). This systematic progression from higher to lower curvature elastic stress showed for the first time that the stimulation of PLC*β*_2_ activity was affected by a fundamental mechanical property of the membrane. The lower the stored curvature elastic stress in the lipid vesicles, the lower the PLC*β*_2_ activity in the presence of GG-Rac2 and GTP*γ*S (i.e. stimulation, Fig. 8).

This new finding suggests that the PLC*β*_2_-GG-Rac2 interaction does indeed have a component of membrane insertion as part of the regulatory mechanism. However, the data collected so far do not allow attribution of the membrane insertion exclusively to one or other of the two proteins (PLC*β*_2_ and GG-Rac2), or to both. Furthermore, the membrane insertion may only be one of the components of a more complex regulatory mechanism. Indeed, the PLC activity measured as a function of the lipid charge and of the stored elastic curvature stress suggests that both electrostatic interactions and insertion might play roles in the regulation of the PLC activity.

### The function of the X-Y linker in the regulatory mechanism of PLC*β*_2_

The data presented here that focus on the function of the X-Y linker fit well with the hypothesis that it possesses an auto-inhibitory function, and are in line with the literature^4^. Indeed, removal of the X-Y linker resulted in an enzyme with higher basal activity, both in the *in vivo* system and in the cell-free system. Thus, in the *in vivo* system, the deletion mutants PLC*β*_2_ (2–803 AA) ∆(470–540 AA) and PLC*β*_2_ (2–803 AA)∆(470–529 AA) showed ca. 1.9- and ca. 4.8-fold higher basal activity, respectively, than w.t. PLC*β*_2_ (2–803 AA). The same trend was observed when comparing the activity of purified w.t. PLC*β*_2_ (2–803 AA) and purified PLC*β*_2_ (2–803 AA) Δ(470–540 AA) in the presence of GDP and GG-Rac2 using the cell-free system.

Based on the data from the cell-free system, where either the electrostatic potential or the stored curvature elastic stress were changed, it is not possible to determine by which mechanism the auto-inhibition is released. It is conceivable that the X-Y linker is repelled by the electrostatic charge of the membrane, and the subsequent conformational re-arrangement of the X-Y linker leads to activation of PLC*β*_2_^4^. However, alternative mechanisms are also compatible with the data, such as partial insertion into the membrane triggering conformational change. Indeed, it cannot be ignored that the activity measurements from the cell-free system indicate that both the electrostatic potential and the stored curvature elastic stress regulate PLC*β*_2_ stimulation, consistent with partial protein insertion.

## Conclusion

The complementary use of biochemical and biophysical approaches led us to a better understanding and characterization of the regulatory mechanism of PLC*β*_2_ as a model of the PLC family. In particular, from this study it emerged that membrane properties modulate PLC activity, which is in line with other recent work on the regulatory mechanism of PLCs^4^,^27^. Even though the regulation of PLC by GTPases has to date been the object of more intense investigation, the regulatory role of the membrane is attracting growing attention. This study provides strong evidence that the membrane plays an integral role in PLC regulation and should be considered alongside regulation of PLC by GTPases. Future studies are expected to extend the current understanding, and to characterize in detail the complementary regulation of PLCs by membranes and proteins.

## Additional Information

**How to cite this article**: Arduin, A. *et al.* Regulation of PLC*β*_2_ by the electrostatic and mechanical properties of lipid bilayers. *Sci. Rep.* **5**, 12628; doi: 10.1038/srep12628 (2015).

## Figures and Tables

**Figure 1:**
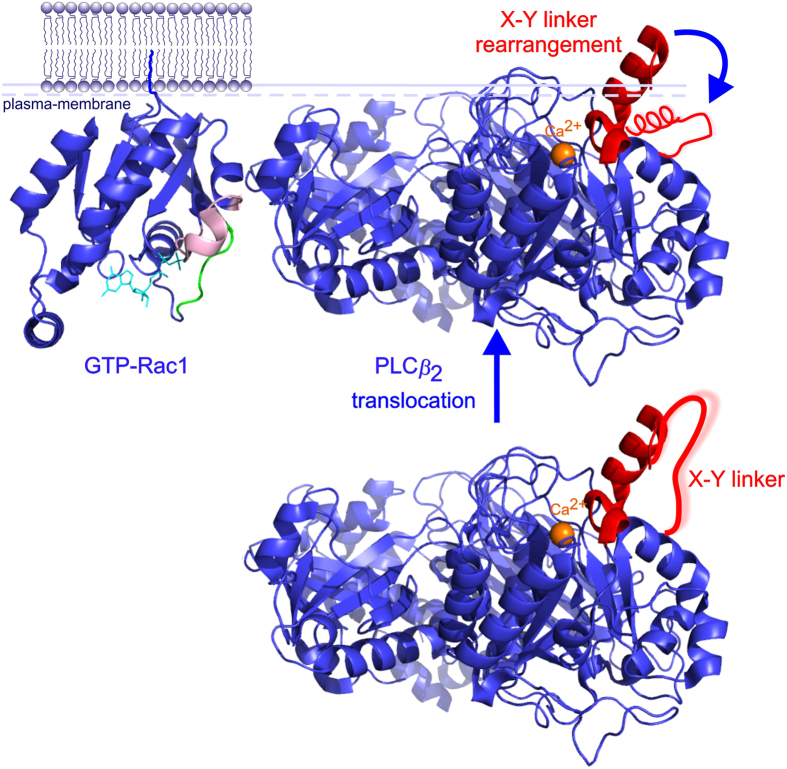
Structures of PLC*β*_2_ obtained in isolation (lower; PDB ID = 2ZKM)^4^ and GTP-bound Rac1 (upper; PDB ID = 2FJU)^5^ illustrating the auto-inhibitory model. Rac1 switch1 is in green, switch 2 is in pink, and the X-Y linker is in red.

**Table 1:** Lipid mixture compositions and corresponding *d* spacings of lamellar phases after hydration in UF water, or cell-free assay buffer (^#^), measured by SAXS.

Lipid mixture	Lipid ratio (mol/mol)	*d* spacing (Å)
DOPE : DOPS : PtdIns(4,5)P_2_	11.2 : 4.4 : 1	121.87+/−0.60
DOPE : DOPS : PtdIns(4,5)P_2_	8.0 : 7.4 : 1	129.21+/−1.45
DOPE : DOPC : PtdIns(4,5)P_2_	11.1 : 4.2 : 1	52.24+/−0.15
DOPE : DOPC : PtdIns(4,5)P_2_	8.0 : 7.0 : 1	55.25+/−0.10
DOPE : DOPC : PtdIns(4,5)P_2_	8.0 : 7.0 : 1^#^	60.81+/−0.55
DOPE : DOPC : PtdIns(4,5)P_2_	4.8 : 9.8 : 1	62.72+/−0.15
DOPC : DMPC : PtdIns(4,5)P_2_	9.8 : 4.9 : 1	64.27+/−0.26
DOPC : DMPC : PtdIns(4,5)P_2_	7.0 : 8.1 : 1	67.92+/−0.32
DOPC : DMPC : PtdIns(4,5)P_2_	7.0 : 8.1 : 1^#^	68.15+/−0.82
DOPC : DMPC : PtdIns(4,5)P_2_	4.2 : 11.4 : 1	62.19+/−0.11

**Table 2:** Biophysical properties of the lipids used in this study: bending modulus (*κ*), spontaneous curvature (C_o_) and cross-sectional area per lipid molecule (A).

Lipid	*κ* (J)	Ref.	C_o_(Å^−1^)	Ref.	A(Å^2^)	Ref.
DOPC	3.7 × 10^−20^	28	0.007	29	72.7	30
DMPC	6.5 × 10^−20^	31	0.025	29	59.6	30
DOPE	4.5 × 10^−20^	28	0.043	29	65	28
DOPS	4.11 × 10^−20^	32	0.0069	32	64.1	33
